# Leveling Up: Evaluation of IV v. PO Linezolid Utilization and Cost after an Antimicrobial Stewardship Program Revision of IV to PO Conversion Criteria within a Healthcare System

**DOI:** 10.3390/pharmacy11020070

**Published:** 2023-04-05

**Authors:** Jessica Jaggar, Kerry O. Cleveland, Jennifer D. Twilla, Shanise Patterson, Athena L. V. Hobbs

**Affiliations:** 1 College of Pharmacy, University of Tennessee Health Science Center, 910 Madison Ave., Memphis, TN 38104, USA; 2 Department of Pharmacy, Methodist University Hospital, 1265 Union Ave., Memphis, TN 38104, USA; 3 College of Medicine, University of Tennessee Health Science Center, 1325 Eastmoreland Ave. #460, Memphis, TN 38104, USA; 4 Department of Pharmacy, Methodist South Hospital, 1300 Wesley Dr., Memphis, TN 38116, USA; 5 Cardinal Health, 13651 Dublin Court, Stafford, TX 77477, USA

**Keywords:** IV to PO, enteral, parenteral, linezolid, cost savings, antimicrobial stewardship

## Abstract

The CDC’s Core Elements of an Antimicrobial Stewardship Program (ASP) lists intravenous (IV) to oral (PO) conversion as an important pharmacy-based intervention. However, despite the existence of a pharmacist-driven IV to PO conversion protocol, conversion rates within our healthcare system remained low. We aimed to evaluate the impact of a revision to the current conversion protocol on conversion rates, using linezolid as a marker due to its high PO bioavailability and high IV cost. This retrospective, observational study was conducted within a healthcare system composed of five adult acute care facilities. The conversion eligibility criteria were evaluated and revised on 30 November 2021. The pre-intervention period started February 2021 and ended November 2021. The post-intervention period was December 2021 to March 2022. The primary objective of this study was to establish if there was a difference in PO linezolid utilization reported as days of therapy per 1000 days present (DOT/1000 DP) between the pre- and post-intervention periods. IV linezolid utilization and cost savings were investigated as secondary objectives. The average DOT/1000 DP for IV linezolid decreased from 52.1 to 35.4 in the pre- and post-intervention periods, respectively (*p* < 0.01). Inversely, the average DOT/1000 DP for PO linezolid increased from 38.9 in the pre-intervention to 58.8 for the post-intervention period, *p* < 0.01. This mirrored an increase in the average percentage of PO use from 42.9 to 62.4% for the pre- and post-intervention periods, respectively (*p* < 0.01). A system-wide cost savings analysis showed projected total annual cost savings of USD 85,096.09 for the system, with monthly post-intervention savings of USD 7091.34. The pre-intervention average monthly spend on IV linezolid at the academic flagship hospital was USD 17,008.10, which decreased to USD 11,623.57 post-intervention; a 32% reduction. PO linezolid spend pre-intervention was USD 664.97 and increased to USD 965.20 post-intervention. The average monthly spend on IV linezolid for the four non-academic hospitals was USD 946.36 pre-intervention, which decreased to USD 348.99 post-intervention; a 63.1% reduction (*p* < 0.01). Simultaneously, the average monthly spend for PO linezolid was USD 45.66 pre-intervention and increased to USD 71.19 post-intervention (*p* = 0.03) This study shows the significant impact that an ASP intervention had on IV to PO conversion rates and subsequent spend. By revising criteria for IV to PO conversion, tracking and reporting results, and educating pharmacists, this led to significantly more PO linezolid use and reduced the overall cost in a large healthcare system.

## 1. Introduction

The CDC’s Core Elements of an Antimicrobial Stewardship Program (ASP) lists intravenous (IV) to oral (PO) conversion as an important pharmacy-based intervention [1]. Additionally, the Infectious Disease Society of America (IDSA) Guidelines recommend implementing ASP IV-PO conversions for both empiric as well as directed therapy to reduce the use of IV catheters and complications, hospital length of stay, the use of outpatient parenteral therapy, and all associated costs [2]. Numerous studies exhibit the benefit of pharmacist-driven IV to PO conversion protocols within the acute care setting, but the literature is limited regarding the revision of such protocols when adherence is low. In this study, we show how we revised the already existing protocol and re-engaged pharmacists in this initiative.

## 2. Materials and Methods

This retrospective, observational cohort study was conducted in a healthcare system composed of 5 adult acute care facilities. The flagship hospital is an academic medical center with 583 licensed beds and an ID pharmacist on staff. The other four hospitals are non-academic, community hospitals that have their own ASPs, but do not have an ID pharmacist on staff. The system ASP led by an ID physician and ID pharmacist reviewed and revised the IV to PO conversion eligibility criteria and implemented it 30 November 2021 (Table 1). All antimicrobials that were identified by the ASP committee as having high bioavailability were included in the pharmacist-driven IV to PO conversion protocol; however, due to the high cost of IV linezolid, this agent was used as a marker for monitoring the rates of pharmacist-driven IV to PO conversion within the healthcare system.

There were no changes to pharmacist daily workflow, as evaluation for IV to PO conversion was already included in the pharmacist daily medication review. Education with the new IV to PO conversion criteria changes was conducted in October 2021 and included a compulsory computerized training module assigned to every pharmacist in the system with an online competency check, which required attestation that pharmacists had completed it. Additionally, optional live presentations were conducted to allow all pharmacists an opportunity to ask any questions. Follow-up emails tracking and reporting results were also sent to the system ASP pharmacy leaders during each quarter in the post-intervention period, and leaders were encouraged to share results with their departments. Results were additionally shared during Pharmacy and Therapeutics committee meetings and minutes. To the authors’ knowledge, there were no changes to the indications for linezolid pre- and post-intervention. 

The primary objective of this study was to determine if there was a difference in PO linezolid utilization reported as days of therapy per 1000 days present (DOT/1000 DP) between the pre- and post-intervention periods. The pre-intervention period was February 2021 to November 2021, and the post-intervention period was December 2021 to March 2022. Secondary objectives included IV linezolid utilization and cost savings. Hospital- and system-level IV and PO linezolid utilization data were collected and evaluated monthly in the post-intervention period. The IV and PO linezolid cost data were measured as the amount spent on each dosage form from monthly pharmacy drug purchase data, with linezolid IV in Dextrose 5% and 0.9% Sodium Chloride piggyback categorized as IV linezolid cost, and linezolid 600 mg tablets and linezolid 100 mg/5 mL oral suspension tracked as PO linezolid cost. At the time of study evaluation, the average wholesale cost for 1 linezolid 600 mg oral tablet was USD 2.00, and the average wholesale cost for 1 linezolid 600 mg intravenous bag was USD 40.02. The standard dosing for linezolid within the healthcare system was the FDA-approved dose of 600 mg Q12 h. Cost and utilization data (DOT/1000 DP) were tracked for the five hospitals individually, and results from the academic versus non-academic hospitals were also evaluated.

## 3. Results

**Figure 1:**
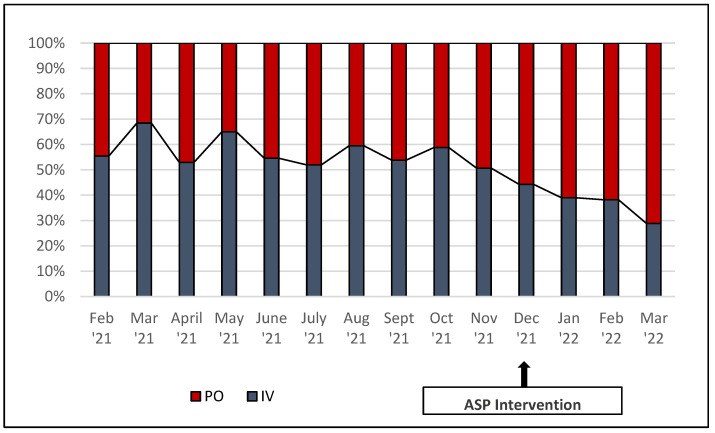
Primary outcome: system linezolid IV vs. PO monthly utilization rates (days of therapy per 1000 days present).

**Figure 2:**
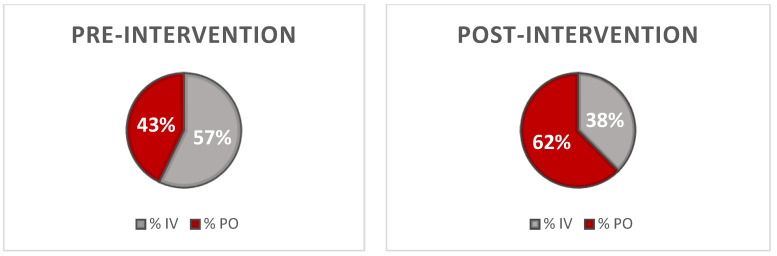
Pre-intervention and post-intervention of IV vs. PO linezolid utilization at the healthcare system: IV = intravenous, PO = oral.

**Figure 3:**
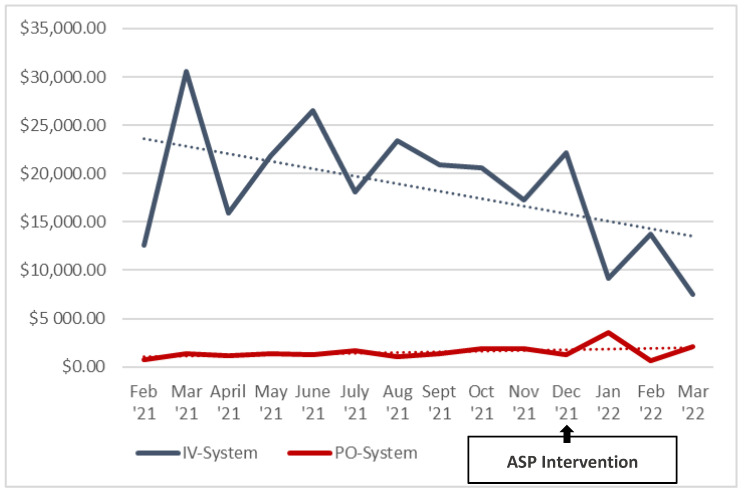
Total cost of IV and PO linezolid for the healthcare system by month. IV = intravenous, PO = oral.

The average DOT/1000 DP for IV linezolid fell from 52.1 to 35.4 in the pre- and post-intervention periods, respectively (*p* < 0.01). Inversely, the average DOT/1000 DP for PO linezolid rose from 38.9 to 58.8 in the pre- and post-intervention periods, respectively (*p* < 0.01, Figure 1). This change reflected a system-wide increase in the average percentage of PO use from 42.9% to 62.4% for the pre- and post-intervention periods, respectively (*p* < 0.01, Figure 2). System-wide, a cost savings analysis revealed average monthly post-intervention savings of USD 7091.34 with projected total annual cost savings of USD 85,096.09 for the system (Figure 3). This result was consistent at both the academic and non-academic hospitals. The pre-intervention average monthly spend on IV linezolid at the academic flagship hospital was USD 17,008.10, which decreased to USD 11,623.57 post-intervention, a 32% reduction. PO linezolid spend pre-intervention was USD 664.97 and increased to USD 965.20 post-intervention. 

**Figure 4:**
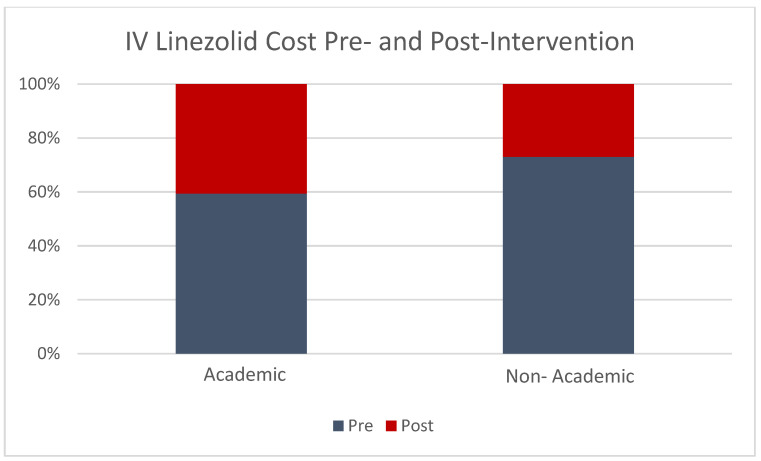
Percent difference in the average monthly cost of IV linezolid in the pre- and post-intervention groups for academic and non-academic hospitals.

The average monthly spend on IV linezolid for the four non-academic hospitals was USD 946.36 pre-intervention, which decreased to USD 348.99 post-intervention, a 63.1% reduction (*p* < 0.01). Simultaneously, the average monthly spend for PO linezolid was USD 45.66 pre-intervention, which increased to USD 71.19 post-intervention (*p* = 0.03). The number of unique patients who received IV linezolid and PO linezolid included in this study was n = 1065, and n = 1068, respectively. Though perhaps not as impressive numerically as the academic facility, the average monthly percentage of IV cost was much lower in the post-intervention group for the non-academic facilities compared to the academic hospital (26% vs. 41% respectively, Figure 4).

## 4. Discussion

Newer evidence supporting the use of PO therapy for complicated infections requiring a long duration of antimicrobial therapy, including the POET and OVIVA studies, combined with low IV to PO conversion rates, spurred our healthcare system’s ASP team to review and streamline the conversion criteria [2,3]. The revised criteria made it easier for pharmacists to convert eligible patients by simplifying the minimum criteria for conversion to PO (Table 1). After pharmacist review, the patient would be converted to PO linezolid if the clinical situation and indication was deemed appropriate. These simplified criteria, combined with updated pharmacist education, monthly data tracking, and reporting to frontline pharmacists all likely contributed to the increased PO linezolid utilization. Subsequently, we were able to show cost savings at a healthcare system level. 

Previous studies have shown that IV to PO conversion programs can increase PO utilization and reduce drug costs [4,5,6,7,8,9,10]. Our study results highlight the positive impact that the revision of an ASP program’s pre-existing IV to PO conversion criteria and protocol can have to decrease IV medication utilization rates and increase PO utilization, resulting in overall decreased costs. Converting from IV to PO can reduce costs by reducing the use of more expensive IV medications, limit human resources needed to compound and administer IV preparations, and eliminate the need for IV catheters and equipment [3]. More importantly, IV to PO conversion programs and ASP interventions have been shown to decrease the hospital length of stay and reduce readmission rates [4,11]. While most studies to date only evaluate the effects of initial implementation of an ASP IV to PO conversion program, there is limited literature that shows improvement in IV to PO conversion rates of antibiotics after pharmacist education and policy updates. Similarly, research has shown that adding a pre-existing conversion protocol along with pharmacist education may increase IV to PO conversion rates. Kan T et al. identified an increase in IV to PO antibiotic conversion rates from 29% to 74% after implementing a clinical decision support tool and providing pharmacist and physician education [12]. Most importantly, the high rates of conversion were sustained at 74% 15 months post-intervention during the follow up period. In this study, however, no changes were made to the eligibility criteria. Other previous studies have shown improvement in IV to PO conversion rates with guideline implementation, checklists, and pharmacist education [13,14]. However, person-based strategies such as education and policies that occur in isolation without changes to criteria or addition of clinical support tools have not shown long-term sustained success. The addition of clinical decision support tools or modifications to criteria may be required to sustain improved rates of IV to PO conversion [12].The authors are unaware of any studies from the US that evaluate the results of improving utilization of conversion criteria at a multi-hospital system level instead of an individual hospital or comparing the effectiveness of an IV to PO conversion criteria and utilization rates at academic vs. non-academic hospitals.

Furthermore, this study showed that while the revision of the conversion criteria as well as tracking and reporting results required the expertise of an ID pharmacist, both the academic and non-academic hospitals were successful in significantly reducing IV linezolid use from the pre- to post-intervention periods. In fact, the average monthly cost percentage for IV linezolid was numerically lower in the post-intervention period for the non-academic hospitals. Unfortunately, researchers were unable to account for possible confounders such as patient acuity that might shunt more patients who met criteria to receive IV to the academic facility.

Additional areas of further research include the revision of conversion criteria for IV medications other than antibiotics, and the resulting cost savings. Authors would also like to evaluate the long-term sustainability of high IV to PO conversion rates after the intervention.

The limitations included that linezolid was the only antibiotic reported in this evaluation. This was for practical reasons, as the ASP team’s bandwidth did not allow for the tracking of all the antibiotics included in the IV to PO conversion policy, and linezolid was identified as the highest priority IV agent. Another limitation is the restricted time frame in the post-intervention period, which was dictated due to a leave of absence by the ID pharmacist at the academic facility who also served as the co-chair of the system ASP. However, due to the significant results seen in the four months post-intervention, the authors would like to explore an expanded study timeframe in the future. Additionally, since the results of this study were based on purchase data, there were no patient-specific data that could inform the clinical impact of the conversion, so we are unable to state whether the updated conversion criteria impacted hospital length of stay or rate of line-associated infections. We were also unable to capture the indications for linezolid or track whether any patients were switched back to IV linezolid after being switched to PO; however, we are unaware of any hospital or healthcare system level guideline changes that would have impacted the indications for linezolid in the pre- vs. post-intervention periods. Our data revealed that “leveling up” on routine ASP initiatives coupled with effective education strategies has the potential to impact antibiotic utilization as well as cost. Revision of IV to PO conversion criteria led to an increase in PO linezolid use and a decrease in IV linezolid spend.

## 5. Conclusions

An ASP intervention that revised the criteria for IV to PO conversion, educated pharmacists, and tracked and reported results led to significantly more PO linezolid use compared to IV and reduced the overall cost in a large healthcare system.

## Figures and Tables

**Table 1:** Criteria for IV to PO Conversion of Antimicrobials.

	Criteria for IV to PO Conversion of Antimicrobials
Pre-intervention	The patient must: Have already received at least 1 day of IV therapy Show some improvement of signs and symptoms of infection, which were present when therapy was started including: Be afebrile (oral temperature < 38 °C) on 2 consecutive occasions Have a white blood cell count (WBC) decreasing and currently < 20,000/mm^3^ Have a functioning GI tract with no known GI malabsorptive disorder, as evidenced by the patient receiving scheduled medications prescribed orally OR tolerating a diet or tube feeding Have an indication for antimicrobial therapy that is NOT one of the following: endocarditis, MSSA/MRSA bacteremia, osteomyelitis, or meningitis
Post-intervention	The patient must: Have a functioning GI tract with no evidence of GI malabsorption, shown by the patient receiving scheduled oral medications or tolerating a diet or tube feeds Be afebrile for at least 24 h (oral temperature < 38 °C)

GI = gastrointestinal, IV = intravenous, PO = oral.

## Data Availability

Third party data. Data were obtained from Methodist LeBonheur Healthcare and are available with the permission of Methodist LeBonheur Healthcare.
